# Hospitalization Experience of Muslim Migrants in Hospitals in Southern Spain—Communication, Relationship with Nurses and Culture. A Focused Ethnography

**DOI:** 10.3390/ijerph17082791

**Published:** 2020-04-17

**Authors:** Fernando Jesús Plaza del Pino, Verónica C. Cala, Encarnación Soriano Ayala, Rachida Dalouh

**Affiliations:** 1 Department of Nursing, Physiotherapy and Medicine, University of Almería, 04120 Almeria, Spain; 2 Department of Research Methods in Education, University of Almeria, 04120 Almeria, Spain; vcc284@ual.es (V.C.C.); esoriano@ual.es (E.S.A.); rachidadalouh@ual.es (R.D.)

**Keywords:** hospitalization, Muslim, communication, culture, nursing, focused ethnography, qualitative methodology

## Abstract

The coast of southern Spain is one of the main entry points for Africans who want to reach Europe; in this area, there is an important immigrant community of African origin, mostly Muslims. The objective of this study is to describe and understand the hospitalization experience of Muslim migrants in public hospitals in southern Spain, especially their relationship with the nurses who care for them. Data were collected from May 2016 to June 2017. This study followed the principles associated with focused ethnography. During data collection, open interviews with 37 Muslim patients were conducted. Three themes emerged from the inductive data analysis: lack of communication with nurses, discriminatory experiences at the hospital and their experience of Islam in the hospital. We conclude that caring for Muslim patients requires specific training not only for nurses but also for other health professionals; existing communication problems must be addressed by establishing the role of the intercultural mediator as an idiomatic and cultural bridge between patients and nurses. In addition, hiring health professionals with migrant backgrounds would help convert hospitals into spaces for intercultural coexistence.

## 1. Introduction

The number of international migrants has increased over time at a much higher rate than some had predicted [1]. Current global estimates indicate that in 2019, there were approximately 272 million international migrants worldwide, equivalent to 3.5% of the world’s population [2]. Migrants leave their countries of origin seeking better access to social services, including health and education and employment opportunities [3]; the migratory process achieves this access [4]. Migratory flows lead to a progressive increase in cultural and religious diversity in recipient countries [5]. Several studies have shown the consequences of cultural and linguistic diversity for the care offered by health services, such as cultural misunderstandings, increased stress and anxiety of professionals or language barrier [6,7,8,9]. The different characteristics of the migrant population can hinder patient access and/or decrease the quality of healthcare received [10], leading to considerations of the foreign patient as complicated [11] and problematic [12]. From the nursing perspective, theories and models have emerged that aim to improve health care for this diverse population [6,13,14,15,16,17,18,19,20].

Until the end of the 20th century, the ethnic-cultural profiles of the health care professionals working in hospitals in Spain and the populations those hospitals serve were largely homogeneous: both comprised Spanish citizens with Catholic backgrounds [7] and health systems were organized “for a culturally, linguistically and socially uniform population” [21]. Although the ethnic-cultural profile of health professionals remains the same, that of users of the public health system has changed substantially; foreigners residing in Spain account for 10.7% of the population, of which 36.3% are European and 22.3% are African [22]. The coast of southern Spain is one of the main entry points for Africans who want to reach Europe [23] using Spain as a transit country [24]. In this area, there is an important African Muslim community, with those of Moroccan nationality comprising the largest group [22]. The fact that in Spain, public health care is free and universal [25] makes migrants potential users of the Public Health System regardless of whether they are documented, although existing data on access to care health care for migrants show that they use fewer health services than the local population [26]. This cultural diversity of the population affects basic aspects of health care [27] and poses a challenge for health professionals, especially nurses, as they are the first line of contact with and assistance to these new users [28]. It is worth asking whether the care provided by professionals fits the needs of these new users and whether this group perceives this care as satisfactory [29]; such issues are even more important to consider given that a high percentage of this population is from the Muslim tradition. The objective of this study is to describe and understand the hospitalization experience of Muslim migrants in public hospitals in southern Spain, especially in terms of their relationship with the nurses who care for them.

## 2. Materials and Methods

### 2.1. Study Design

A qualitative study was designed following the principles associated with focused ethnography [30] and maintaining an approach located in the interpretive paradigm. Focused ethnography is appropriate for health research because it can provide answers to specific questions before research is conducted. It is characterized by its focus on a specific community, organization or social phenomenon within a given context, requires a limited number of participants and is useful in health care services [31]. In our case, it was used to explore the experiences of Muslim immigrants in public hospitals in the south of Spain.

### 2.2. Participants and Context

The study informants are Muslim migrant patients (MP) seen at three public hospitals in southern Spain, two county hospital and one regional hospital. Spain. Intentional sampling was conducted to select participants from different age groups, genders, nationalities, lengths of stay in Spain, levels of knowledge of Spanish and so forth, to help ensure extensive exposure to the specific environment. All participants had at least one hospital stay of no less than three days in a public hospitalwithin the month prior to the interview.

Participation was voluntary. The principal investigator recruited the participants, although there was collaboration from nurses, who facilitated contact with patients who met the inclusion criteria. Two participants withdrew from the selection process when their city of residence changed.Data were collected from May 2016 to June 2017.

When 37 participants had been interviewed, topics began to be repeated; at that point, the researchers felt that data saturation had been achieved and data collection was completed.

The study involved 37 MPs, among which Moroccans predominated, with 20 individuals. The mean age was 34.4 years. Seventeen women and 20 men participated. The characteristics of all the informants are presented in Table 1.

### 2.3. Data Collection

Data collection was carried out through open interviews following a guide with open questions to facilitate in-depth discussion of the topics of interest, we ask questions such as the treatment received, the respect and knowledge of their Muslim customs and traditions by the nurses or the situations experienced in the hospital.

It was agreed with the informants that the interviews were conducted in their own homes to ensure that the environment was adequate for facilitating the sincere expression of feelings and emotions. The average duration of the interviews was forty minutes and they were conducted by the research team in Spanish, Arabic and several African languages from Senegal and Mali (Wolof, Bambara and Mandaic) with the participation of interpreters, depending on whether the participant spoke the Spanish language. The interviews were recorded with the participant’s permission.

### 2.4. Management and Data Analysis

The interviews were transcribed and reviewed by the interviewers. In the case of the interviews conducted in languages other than Spanish, they were transcribed in the language of the interview and subsequently translated into Spanish. The data were stored, managed, classified and sorted with the aid of the qualitative data analysis software ATLAS-ti 8.0 (Berlin, Germany). Open coding of the interview data was performed, whereby we identified themes and key patterns in order to generate the first categories to work with.

### 2.5. Ethical Considerations

The Research Ethics Committee in Almería, CEI, approved the research protocol (protocol number: 52/2015). To ensure anonymity and confidentiality, a code was assigned to each participant. In all cases, a written informed consent form was provided and explained.

## 3. Results

After initially identifying 93 codes in the data, the more prominent codes were grouped into key categories. A more detailed analysis allowed the identification of three main thematic categories (Table 2).

### 3.1. MPs Facing a Lack of Communication from Nurses

This category encompasses all issues related to communication/lack of communication between informants and nurses and the efforts they make to overcome the language barrier.

#### 3.1.1. The Importance of Communication. Actively Seeking Tools to Overcome the Lack of Communication

MPs are aware of the importance of knowing the language and being able to communicate with health care providers, as these abilities will shape the therapeutic relationship formed in the hospital.

When you are with the nurse, she speaks on one side and you speak on the other. You can only point to where it hurts but you cannot explain; she will not know what happens to you.

(M17)

Not speaking the language has an effect. For instance, I have a good command of Spanish and I can socialize more with people, I ask questions and so on. As a result, they spend more time with me; they see what I am like. However, if someone cannot speak the language, he will be isolated in his own world and people will say and this guy, what is he doing? What is he thinking? And they start judging him because he is quiet. I think this aspect has a strong influence on discrimination.

(M12)

Using informal translators. Given the lack of communication with nurses due to the language barrier, MPs turn to their children, partners, friends or roommates because there are no professional translators offered by the hospital (in almost all the cases studied):

No, I did not say that; it was my sister who spoke. The husband went with her.

(H6)

Those who came to visit me, they translated the things that the doctor said that I did not understand.

(H10)

MPs are aware that the lack of rigor in the transmission of clinical information can produce erroneous translations of diagnoses, treatment and care guidelines and can cause misunderstandings and other negative consequences, such as loss of the confidentiality of clinical information or the risk of manipulation of transmitted information, especially when information is conveyed by informal translators close to the patient.

Communication is not easy, even with a friend who translates, because those who do not work in the hospital do not know how to explain things well.

(H13)

In some cases, the professionals themselves resort to informal translators to overcome the language barrier with their MPs.

In the hospital, the nurse told me to look for a fellow countryman who speaks good Spanish, that she will come back and he can explain everything to her.

(H10)

Searching for other solutions to lack of communication. Both professionals and MPs attempt to overcome the language barrier by seeking solutions to the lack of formal and informal translators using pictograms, non-verbal language tools or documentation in other languages, such as Arabic or French:

There was no problem; it was all drawn.

(M10)

Yes, he spoke like you; he understood, like I do now. He spoke slowly and he gestured to me.

(H2)

When I left, they gave me a book in Arabic and Spanish about the child’s things.

(M8)

#### 3.1.2. When there is no Translation, the Rights of the Patient are Violated. Professionals only Complete the Tasks and Leave

MPs who do not speak Spanish have commonly been hospitalized without any access to someone who can translate for them, which violates their right to be informed about their process, treatment and prognosis in their language and to make their own decisions.

(In the delivery room)At first, I told them that I do not understand anything and they left me as I was. They have done their job but they have not explained anything to me, as I do not speak Spanish and I do not understand anything. That bothered me a lot.

(M3)

They told me to sign but I do not know what I put on paper.

(H14)

We found informants who tell us about nurses who do not care about the existence of a language barrier, who do not try to overcome it and simply do not communicate with their MPs, which shows a lack of commitment to the quality of their care and deficient professionalism.

When the nurses talk to you and they see that you do not speak at all, then they do their job and leave.

(H8)

MPs state that they have found nurses who, upon identifying them as foreigners, have assumed that there is a language barrier and have not attempted to communicate with them, even though they speak Spanish.

She does not talk to me; she does not ask me about the stitches and she hurts me. I scream and she stops.

(M8, patient who speaks Spanish)

In many cases, the informants indicated that they have not insisted on translation because they did not feel translation was *necessary* because of *the trust* they place in the health system and its professionals. This trust allowed them to make assumptions about treatments, diagnostic tests or surgical interventions without being informed.

The truth is that I did not understand anything but I trusted that what they did was the best for me.

(H14)

When I have felt ill and I have been in the hospital, I have trusted the whites, the Spaniards.

(H16)

### 3.2. Discriminatory Relationships in the Hospital

#### 3.2.1. No Problems with Interacting

The assessment by MPs of their relationship with nurses was mostly positive, highlighting above all the nurses’ professionalism and the trust they convey to the MPs. The MPs also highlighted the care they received and the treatments available in the hospital centers.

Regardless of being poor or rich, you are in the hands of professionals. (…) I trusted that what they did was the best for me.

(H2)

I called and got what I needed. They gave me many medicines that made me heal well.

(M9)

#### 3.2.2. Softening Discrimination. Satisfaction with Treatment and Justification of Discrimination

There were few participants who had problems with the nurses or who complained about the treatment received, although several did describe situations in which they experienced discrimination.

Yes, a nurse disrespected me and called me mora. I told her, “Look, my mother killed two lambs to call me... (the MP’s name).” I fought, yes, yes, because she said, “That one, the mora”; she said it like that (makes a gesture of contempt) and it made me angry because that cannot be.

(M5)

MPs who had negative experiences with nurses tended to downplay and even justify discriminatory behaviour by minimizing it and treating it as something exceptional, although their comments described repeated discriminatory behaviours.

Some nurses seem to be fed up with treating people; they have a lot of work and that is why sometimes they can respond poorly.

(H3)

Some things they do without realizing it.

(M14)

This nurse, it was noticeable, did not approach me. I preferred to wait until another one came by when I was in pain because if she came, she would talk to me badly.

(M2)

Some MPs avoided making nurses responsible for their discriminatory behaviour and even blamed the patient:

I understand that the majority of Moroccans who pass through the hospital come from farmhouses, many of them without water and arrive at the hospital in a state... a little dirty and the nurse is a little disgusted by people who arrive like this.

(H5)

### 3.3. Living One’s Own Culture. Islam in the Hospital

This topic encompasses categories related to the cultural and/or religious aspects of MPs and sometimes it is difficult to discern between religious practices and specific traditions. Therefore, we will approach the practices and beliefs of the informants through their testimonies without trying to discuss whether they are purely religious practices.

#### 3.3.1. Variability in Practices

The enormous variability in the traditional-religious-cultural practices of our informants is noteworthy. Not all people from a Muslim tradition practice religion in the same way and some of our participants claimed not to believe or practice religion, despite having been educated and culturalized in that tradition.

Regarding daily prayer, one of the five pillars of Islam, our informants highlighted the diversity of experiences within the hospital. What is striking is that despite the great importance of prayer within Islam, many of the MPs stated that they do not pray regularly:

I am a Muslim and I do Ramadan and all that, although I do not pray or go to the mosque.

(H3)

There are Muslims who pray; there are Muslims who do not pray.

(M13)

One of the most well-known characteristics of the Muslim religion is food regulations. Islam prohibits the consumption of certain foods and substances, such as pork and alcohol.

It is hospital food; it is not the food from your home. They bring you food. You are not obligated to eat it. You eat. I did not like it but you have to eat.

(H7)

Among the most common stereotypes about Muslims is the inequality between men and women. The men are considered to be sexist and the women are believed to be submissive to them, which materializes as a marked division between the genders in daily life. For this reason, we decided to ask the MPs about their preferences for being treated by health professionals of the same gender and there was variability on this issue; only a minority said emphatically that they preferred to be treated by professionals of the same gender. Others expressed a desire to be treated by personnel of the same gender but without requiring it or refusing to be treated by the opposite gender:

No, I do not care. As long as they treat me well… what will you do? It is fine.

(M5)

For women, we think that a woman will be better than a male doctor. […] I am afraid to take my wife and that it be a male doctor. That may be a little closed-minded, right?

(M7)

The use of the hijab or Islamic veil is one of the most notorious signs that a woman belongs to the Muslim religion [32]. This practice has generated controversy in several European countries as it isconsidered a sign of the submission of women to men, although it can be justified as a religious or cultural custom or as a symbol of belonging to the community and an important link between women and their families. Despite the debate generated by this issue, we did not find testimonies that revealed problems with wearing a veil during hospital admissions.

My religion says to do it but each one is free to do what she wants. As a Muslim, I like to wear it.

(M3)

I wear the veil some days and not others. For example, when I come to the hospital, I like to wear it.

(M11)

We found comments that do not support the behaviors that are considered more radical, which consider that traditions or beliefs that can be interpreted by the host society as closed-off and that can hinder integration:

If you wear the burqa, you do not see your eyes. As Muslim, we would support it if that practice disappeared.

(H2)

#### 3.3.2. Religion and Traditions are in the Background at the Hospital

Many of our MPs put the need to heal above religious factors and said they adapt their practices and beliefs to the hospital environment, leaving them in the background until they leave the hospital and can return to their usual practices.

About prayer:

No, not in the hospital. When it was over, the operation, yes, I prayed at home.

(H6)

On care by professionals of the opposite gender:

If you are sick, you have to be cured. If it is a man, you have to see him; it is necessary. Childbirth is something natural and “merciful”; you cannot be embarrassed.

(M16)

Or about the use of the hijab:

At birth, they took my headscarf and gave me a cap that was attached to my nightgown, which was fine with me and after I gave birth, I put my headscarf back on.

(M3)

#### 3.3.3. Nurses and the Culture of their MPs

In reference to the perceptions that the MPs had regarding the attitudes and behaviors of nurses with respect to the MPs’ religion and traditions, it is worth noting that in many cases, the professionals presuppose the religious affiliation of these patients based on their country of origin or name:

It seems to me that they have suspected that I am Muslim. I think they think that all black people are Muslims. (

H2)

The MPs noted with some frequency that they were not asked about their religion or whether they followed a special diet, although in other cases, there was information exchange between the nurse and her patient, although it was rare.

They have not asked about these things. This point should be included.

(H15)

When she came the first time, she asked me, “What food do you want?”

(H4)

Regarding the perceptions of the nurses’ cultural knowledge about Islam and their interest in addressing the MPs’ traditions and beliefs, the MPs expressed that they perceived a certain disinterest.

I think not, that they do not show interest in finding out, in knowing, talking about that, about diet, about everything.

(M6)

Some do not understand the cultural things and do not want to understand.

(H3)

#### 3.3.4. We are Less Different than it Seems

Many MPs made reference in their comments to the cultural similarities that they encountered with the culture and inhabitants in the study area, Andalusia. In particular, the MPs of Moroccan origin pointed out the importance of the family and the type of social relations that are established.

For the Moroccans, the family is always with the patient for what they need. That is the same as you; the Spanish are the same as us.

(H4)

We like to be in the street and talk to neighbors; we are less different than it seems.

(M4)

### 3.4. What Improvements would you like to see in Hospitals?

In all the interviews, the last minutes were dedicated to letting the MPs suggest aspects of the hospital environment that could be modified or improved to make them feel better served and culturally respected. They highlighted the lack of requirement when they presented their proposals for improvement; in most cases, they put the need to restore health above anything else and did not consider their suggestions important.

I do not have anything to say. I was sick and I went to the hospital to heal and they healed me. That is what matters; nothing more.

(H7)

I am not the one who should say that. What can I say?

(M10)

Some areas for improvement referred to the need to have a place for prayer. The MPs were aware of the existence of a Christian chapel at the hospital and suggested the creation of a smaller space to pray.

Something can be improved: there is a church in the hospital, [...] and I asked him if there was a mosque and he told me that there was only a church. If they create a place, even if it is two meters by two, where the door can be closed and it can be quiet….

(H19)

In terms of solving communication problems, the MPs proposed the need for translator-mediators in the hospital environment and defined the characteristics that they should have; specifically, they should be not only linguistic translators but also cultural mediators between health professionals and MPs.

I would like to have an official interpreter from the hospital who could explain to me well what I suffer from and how I have to take care of myself… that knows my culture and about the medicine.

(M2)

There were also comments directed towards professionals referring to aspects such as the treatment of migrants or the lack of cultural training. These comments emphasized the need to eliminate discriminatory behaviors and provide cultural training.

Improve humane treatment, so that they do not see us as something strange... that they see us as like anyone else, that they deepen their understanding of the treatment given to immigrants.

(H2)

To know if the Muslim is bad or good, one must study religion.

(H18)

Other improvements are similar to those of any other public health user, which is interpreted as a sign of normality in the use of the health services by many MPs. Such issues include attendance time, shortages of staff and wait time in emergencies.

The first thing is time. They are always in a hurry. [...] Patience... patience for both - the staff and those of us who are sick.

(H3)

The same nurse must attend too many. They need to hire more.

(M15)

## 4. Discussion

The purpose of this study was to describe and understand the experience of MPs hospitalized in public hospitals in southern Spain. We identified several important issues, such as communication problems, the relationship with nurses and experiences with their habits and traditions in the hospital context; these issues present barriers to accessing quality health care [33,34].

MPs are aware of the importance of adequate communication with the nurses who care for them and the language barrier highlighted by this study has also been widely described in the literature as one of the main challenges to effective access to health care for migrants [33,35,36,37,38]. Communication between nurses and patients is a challenge not only in terms of language but also in the way in which culture can affect the way patients express themselves [35].

The need to overcome the language barrier results in the use of informal translators - people without knowledge in the field of health and, in many cases, without a good knowledge of Spanish. Working with informal translators can be even more dangerous than completely dispensing with interpretation [37,39], since erroneous translations of diagnoses, treatments and care can occur, causing misunderstandings and the loss of confidentiality of clinical information [40]. However, the hiring of bilingual interpreters does not guarantee culturally sensitive attention, either, since they may lack cultural sensitivity when communicating with patients [35,41]. Translation in the field of health requires expert knowledge and skills acquired through training and practice [35].

It is striking that when nurses in situations of communication barriers did not seek solutions, their lack of willingness to communicate or make use of translation tools was contextualized within a system in which human and material resources are reduced. On the other hand, the patients did not demand translation to obtain information about their health, as is their right. The explanation for this resignation to a lack of communication is the trust the MPs have in the public health system and its professionals. This positive view leads them to accept treatments about which they are not informed, sign consent for diagnostic tests or interventions without translation or be discharged without understanding the guidelines they should follow. Non-translation denies the patient the chance to participate in clinical decisions that affect them and can have unforeseeable consequences for both the patient (not knowing their diagnosis, prognosis, treatment or care requirements after discharge from the hospital) and those around them (as in the case of patients with infectious-contagious diseases such as tuberculosis, hepatitis C and HIV); disinformation will prevent patients from taking measures to prevent contagion in their environment, which can lead to public health problems [34].

The MPs’ positive views of the public health system and its professionals, despite experiencing discrimination [33], differs from the findings of other studies [42,43]. The patients tended to minimize and justify unprofessional behaviors, even blaming themselves for nurses’ inappropriate behaviorstoward them. The confirmation of perceived discrimination indicates that the interaction between MPs and their care providers presents problems [44] that should be analyzed in greater depth.

Our data show that not all people of Muslim origin practice religion in the same way. In general, the need to regain health by following some pillars of Islam comes first. The reality of MPs is very different from the stereotypical view of them as a homogeneous collective in which all its members interpret religion in the same way and put religious precepts before other aspects of life [45,46].

Regarding the MPs’ perception of nurses’ respect for their beliefs and traditions, the MPs perceived disinterest in learning about their culture and religion. The lack of intercultural knowledge complicates the therapeutic relationship with patients [47]. The lack of diversity in the ethnic origins of health personnel in hospitals in southern Spain is an obstacle in the provision of intercultural care and the solution may be to incorporate migrants into health services [48,49] to ensure culturally competent care [35], in addition to providing undergraduate and continuing intercultural training for nurses [50,51] and information about the rights of migrants to health care [52].

### 4.1. Limitations

We ensured that our sample was not homogenous, and that great diversity was present among the participants in terms of their personal characteristics, which means that our findings are not generalizable to the entire collective of MPs; nonetheless, our findings can provide insight into the realities that are experienced daily in the context of this research. Additionally, it was not possible to perform observations in the context investigated since the hospital environment is a sensitive area where preserving patient privacy is a priority.

It is necessary to investigate the experience of MPs in primary care to detect problems that may exist in this area.

### 4.2. Implications for Practice

Caring for foreign patients, in our case those from the Muslim tradition, in a way that guarantees culturally adapted care requires specific training not only for nurses but also for other health professionals organized from the hospitals themselves.

The hiring of health professionals from migrant backgrounds will help convert hospitals into spaces of intercultural coexistence.

The communication problems that MPs experience in the hospital environment must be addressed with the implementation of intercultural mediators, who serve as idiomatic and cultural bridges between patients and nurses.

## 5. Conclusions

This study presents an emic perspective on the experiences of MPs in hospitals in southern Spain. The findings highlight that there are communication problems between MPs and health personnel that are still pending, that discriminatory and unprofessional behaviors are observed in relationships with nurses and that cultural knowledge of MPs is scarce.

Hospitals do not provide solutions to the communication problems of MPs; they have to use informal translators with negative consequences for them.

The MPs’ perceptions of the Spanish health system and their trust in its professionals is very high, which is a strength of the system itself for this reason they do not require translation and downplay the discriminatory treatment they receive.

The variability in the religious practices of the MPs and the placement of health recovery above the precepts of religion are at odds with the stereotypes in Western societies of Muslims as a homogeneous group in which all its members interpret the religion the same way and for whom religious precepts take precedent over all other aspects of life.

## Figures and Tables

**Table 1:** Characteristics of the informants.

Code	Gender	Interview Language	Age	Country of Birth	Speaks Spanish	Administrative Situation	Religious Beliefs/Practices
**H1**	Male	Spa-Fr	39	Mali	Yes	Undocum	Practicing
**H2**	Male	Wolof	34	Senegal	No	Undocum	Practicing
**H3**	Male	Spanish	43	Morocco	Yes	Resident	Believer
**H4**	Male	Spanish	43	Morocco	Yes	Resident	Practicing
**H5**	Male	Spanish	52	Morocco	Yes	Community	Practicing
**H6**	Male	Spa-Arab	53	Morocco	Knows some	Resident	Practicing
**H7**	Male	Spa-Port	48	Guinea-Bissau	Yes	Resident	Believer
**H8**	Male	Arabic	39	Morocco	No	Undocum	Practicing
**H9**	Male	Spanish	25	Mali	Knows some	Undocum	Practicing
**H10**	Male	Arabic	33	Morocco	Knows some	Resident	Practicing
**H11**	Male	Spanish	33	The Gambia	Yes	Undocum	Believer
**H12**	Male	Spanish	31	Morocco	Yes	Resident	None
**H13**	Male	Wolof	26	Senegal	No	Undocum	Believer
**H14**	Male	Wolof	22	Senegal	No	Undocum	Practicing
**H15**	Male	Spanish	36	Morocco	Yes	Resident	Believer
**H16**	Male	Bambara	21	Mali	No	Undocum	Believer
**H17**	Male	Arabic	40	Morocco	Knows some	Resident	Practicing
**H18**	Male	Spanish	33	Mali	Yes	Community	Believer
**H19**	Male	French	20	Guinea (Conakry)	No	Undocum	Practicing
**H2O**	Male	Arabic	32	Morocco	Knows some	Resident	Practicing
**M1**	Female	Spanish	35	Guinea (Bissau)	Yes	Resident	Practicing
**M2**	Female	Mandaic	32	Guinea (Bissau)	No	Community	Believer
**M3**	Female	Arabic	32	Morocco	No	Undocum	Practicing
**M4**	Female	Spanish	28	Morocco	Yes	Resident	Practicing
**M5**	Female	Spanish	58	Morocco	Yes	Undocum	Believer
**M6**	Female	Spanish	37	Spain (Melilla)	Yes	Community	None
**M7**	Female	Spanish	38	Palestine	Yes	Resident	Practicing
**M8**	Female	Spanish	38	Morocco	Knows some	Resident	Believer
**M9**	Female	Spa-Arabic	22	Morocco	Yes	Resident	Practicing
**M10**	Female	Spa-Arabic	33	Morocco	Knows some	Resident	Practicing
**M11**	Female	Spa-Arabic	35	Morocco	Knows some	Resident	Practicing
**M12**	Female	Spanish	22	Algeria	Yes	Undocum	Practicing
**M13**	Female	Spanish	43	Morocco	Yes	Resident	Practicing
**M14**	Female	French	41	Senegal	Knows some	Resident	Practicing
**M15**	Female	Spanish	38	Senegal	Yes	Resident	Practicing
**M16**	Female	Spa-Arabic	31	Morocco	Knows some	Resident	Practicing
**M17**	Female	Arabic	33	Morocco	No	Undocum	Believer

**Table 2:** Thematic categories, themes and codes.

Thematic categories	Themes	Codes
MPs face a lack of communication from nurses	The importance of communication. Actively seeking tools to overcome the lack of communication	Using informal translators. Seeking other solutions to the lack of communication
	When there is no translation, the rights of the patient are violated. Professionals only completing tasks and leaving	Nurses who do not care. Lack of professionalism. If you are a foreigner, you do not speak. Information is not necessary because of trust in the health system and its professionals
Discriminatory relationships in the hospital	No problems when interacting	Acknowledgement of the care and available resources
	Softening discrimination. Satisfaction with treatment and justification of discrimination	Justifying discriminatory behavior. Blaming migrants. Minimizing discrimination. Reasons for discrimination
Living one’s own culture. Islam in the hospital	Variability in practices	Prayer
	Religion and traditions in the hospital are in the background	Food
		Different care by gender
		The hijab
	Condemnation of radical behavior	
	Nurses and the culture of their MPs	Nurses presuppose religious affiliation. They do not ask about patients’ cultural patterns. From ignorance to cultural commitment
	We are less different than it seems.	
Improvements the participants would like to see in hospitals	No requirement	Having a place of worship. Translators/cultural mediators. More cultural knowledge by nurses. Same treatment as any other user
